# Peer Review of “Predicting Health Disparities in Regions at Risk of Severe Illness to Inform Health Care Resource Allocation During Pandemics: Observational Study”

**DOI:** 10.2196/25572

**Published:** 2020-12-02

**Authors:** Ross Gore

**Affiliations:** 1 Virginia Modeling, Analysis and Simulation Center Old Dominion University Norfolk, VA United States

**Keywords:** coronavirus, SARS-CoV-2, pandemic, socioeconomic status, predictive model, health care resource allocation

*This is a peer review submitted for the paper “Predicting Health Disparities in Regions at Risk of Severe Illness to Inform Health Care Resource Allocation During Pandemics: Observational Study.”*

## Round 1 Review

### General Comments

This study [1] proposes a repeatable modeling process to identify regional population centers with COVID-19 vulnerability using linear regression. The work is validated using data from states with high population densities, with New York, New Jersey, Connecticut, Massachusetts, Louisiana, Michigan, and Pennsylvania showing the strongest predictive results.

### Specific Comments

#### Major Comments

- The authors describe their stepwise linear regression process in the Methods section. However, I would like to see more detail (probably supplied as supplementary material) on the trendline and scatterplot analysis to identify those variables most correlated with COVID-19 mortalities. It seems a more comprehensive cross-correlational analysis would be appropriate here; perhaps the trendline and scatterplot analysis is sufficient, but it is impossible to tell without additional details and results being provided.
- The paper would be improved if it was grounded by more analytical background material with references. I think a review of related epidemiological models applying stepwise regression in a similar model would help readers buy into this approach and use/cite the authors’ model. This is not my area of expertise but some suggestions include:
  - Thomson MC, Molesworth AM, Djingarey MH, Yameogo KR, Belanger F, Cuevas LE. Potential of environmental models to predict meningitis epidemics in Africa. Trop Med Int Health. 2006 Jun;11(6):781-8. doi: 10.1111/j.1365-3156.2006.01630.x. PMID: 16771998.
  - Chung WM, Buseman CM, Joyner SN, Hughes SM, Fomby TB, Luby JP, Haley RW. The 2012 West Nile encephalitis epidemic in Dallas, Texas. JAMA. 2013 Jul 17;310(3):297-307. doi: 10.1001/jama.2013.8267. PMID: 23860988.
  - Moncayo AC, Edman JD, Finn JT. Application of geographic information technology in determining risk of eastern equine encephalomyelitis virus transmission. J Am Mosq Control Assoc. 2000 Mar;16(1):28-35. PMID: 10757488.
  - Yu, HYR, Ho, SC, So, KFE and Lo, YL. The psychological burden experienced by Hong Kong midlife women during the SARS epidemic. Stress and Health. 2005;21(3):177-184. doi.org/10.1002/smi.1051
- I commend the authors for the validation of their model, but I believe a section describing the model limitations should be added. Specifically, I’d like to see a set of conditions describing what the model is unable to do, or under what conditions its uses are invalid. I assume this list would include:
  - The model is only valid within the range of the data that have been observed thus far.
  - The model would only need to be revalidated for each additional state not included in the Results section for which it is applied.

In addition, I think the authors should note that even though they checked for correlation between subcomponents of the CDC Social Vulnerability Index and COVID-19 mortalities, that other data sets at the FIPS level (ie, American Community Survey data and census data) might yield different results. In particular, there may be many variables correlated with one another that correlate with COVID-19 mortalities. GGore et al [2] showed that it can be very difficult to tease apart specific population demographic measures at a granular level (ie, city or FIPS) into a linear regression model since so many of these variables correlate highly with one another. Making this point and citing the paper would help establish the context under which the model was built and the conditions in which it is valid.

#### Minor Comments

- In the replication crisis error, an anonymized version of the data provided in the paper, the scripts used for analysis, and the scripts used to create the figures for the paper need to be provided to both the reviewers and to the readership. This ensures a completely transparent analysis and makes the authors’ paper significantly more impactful as other researchers can build off (and cite) the paper.
- Figures 2 and 3 use a contrast between red and green to convey meaning. As many as 8% of men and 0.5% of women are affected with the common form of red-green color blindness that creates no contrast between the two colors. The figure would be improved if a different color choice is used. There are numerous options that offer superior readability [3] .

## Round 2 Review

My comments have been addressed; the paper is now suitable for publication.

## References

[1] Fusillo T (2020). Predicting Health Disparities in Regions at Risk of Severe Illness to Inform Health Care Resource Allocation During Pandemics: Observational Study. JMIRx Med.

[2] Gore RJ, Diallo Saikou, Padilla Jose (2015). You Are What You Tweet: Connecting the Geographic Variation in America's Obesity Rate to Twitter Content. PLoS One.

[3] somersault18:24.

